# Phenotypic alterations in liver cancer cells induced by mechanochemical disruption

**DOI:** 10.1038/s41598-019-55920-2

**Published:** 2019-12-20

**Authors:** Hakm Y. Murad, Emma P. Bortz, Heng Yu, Daishen Luo, Gray M. Halliburton, Andrew B. Sholl, Damir B. Khismatullin

**Affiliations:** 1 Department of Biomedical Engineering, Tulane University, New Orleans, LA USA; 2 Tulane Institute for Integrative Engineering for Health and Medicine, Tulane University, New Orleans, LA USA; 3 Department of Pathology and Laboratory Medicine, Tulane University, New Orleans, LA USA; 4 Tulane Cancer Center, Tulane University, New Orleans, LA USA

**Keywords:** Cancer therapy, Hepatocellular carcinoma

## Abstract

Hepatocellular carcinoma (HCC) is a highly fatal disease recognized as a growing global health crisis worldwide. Currently, no curative treatment is available for early-to-intermediate stage HCC, characterized by large and/or multifocal tumors. If left untreated, HCC rapidly progresses to a lethal stage due to favorable conditions for metastatic spread. Mechanochemical disruption of cellular structures can potentially induce phenotypic alterations in surviving tumor cells that prevent HCC progression. In this paper, HCC response to mechanical vibration via high-intensity focused ultrasound and a chemical disruptive agent (ethanol) was examined *in vitro* and *in vivo*. Our analysis revealed that mechanochemical disruption caused a significant overproduction of reactive oxygen species (ROS) in multiple HCC cell lines (HepG2, PLC/PRF/5, and Hep3B). This led to a decrease in cell viability and long-term proliferation due to increased expression and activity of death receptors TNFR1 and Fas. The cells that survived mechanochemical disruption had a reduced expression of cancer stem cell markers (CD133, CD90, CD49f) and a diminished colony-forming ability. Mechanochemical disruption also impeded HCC migration and their adhesion to vascular endothelium, two critical processes in hematogenous metastasis. The HCC transformation to a non-tumorigenic phenotype post mechanochemical disruption was confirmed by a lack of tumor spheroid formation *in vitro* and complete tumor regression *in vivo*. These results show that mechanochemical disruption inhibits uncontrolled proliferation and reduces tumorigenicity and aggressiveness of HCC cells through ROS overproduction and associated activation of TNF- and Fas-mediated cell death signaling. Our study identifies a novel curative therapeutic approach that can prevent the development of aggressive HCC phenotypes.

## Introduction

The heavy, dual blood supply to the liver parenchyma creates favorable conditions for hematogenous dissemination of cancer cells, leading to rapidly progressing liver cancer. Liver failure due to intrahepatic tumor progression and/or metastases from the liver to other organs severely limit treatment options. This makes liver cancer one of the deadliest cancers worldwide, with a mortality rate of 788,000 deaths per year^1–3^. The incidence rate of hepatocellular carcinoma (HCC), the most common form of liver cancer, continuously increases due to excessive alcohol consumption, obesity, diabetes, and hepatitis^4–7^. Most HCC patients are asymptomatic through the intermediate stage, characterized by multifocal or single >5 cm nodule tumors^8^. At this stage, their treatment is limited to palliative therapies such as TACE that merely extend patient life up to 1.5 years^9^. Minimally invasive curative strategies for large liver tumors that can prevent HCC progression to its lethal, advanced stage are critically needed.

The existing nonsurgical curative approaches, such as radiofrequency ablation (RFA) and percutaneous ethanol injection (PEI), are effective only for the treatment of single <3 cm nodules^10,11^. High-intensity focused ultrasound (HIFU) is a novel radiation-free non-invasive ablation modality. It is less susceptible to the heat-sink effect (heat transfer away from the tumor by the flow of blood), which is one of the reasons for incomplete tumor ablation after RFA and other thermal methods^12^. Standalone HIFU has been successfully used for ablation of small tumors in the prostate and thyroid^13–15^. Our recent studies indicate that HIFU has great potential as an adjuvant/enhancer of PEI and targeted therapies such as tyrosine kinase inhibitors^16–18^. In recent clinical studies^19,20^, a combination of HIFU with transcatheter arterial chemoembolization (TACE, alternative approach to PEI in which embolic agents are used to block blood circulation in the tumor) was shown to be more effective than standalone TACE for treatment of <5 cm HCC tumors. All these demonstrate that HIFU works synergistically with chemical agents to induce tumor destruction. It should be noted that TACE influences the conditions where tumor cells grow, but its ability to directly change the cell phenotype, alone or when combined with HIFU, is questionable.

HIFU induces intense vibration of the cell, which causes disruption of the cellular membrane, cytoskeleton, and other structures. This leads to ROS-producing exogenous stresses^21^. Cancer cells exposed to PEI also experience exogenous stresses resulting in elevated ROS production^22^. Chemical drugs that produce large amounts of ROS are known to reduce proliferative and invasive potentials of cancer cells. ROS mediates cancer cell killing via TNF- and Fas-mediated apoptosis^23,24^ and triggers cell autophagy^25^. We hypothesize that mechanochemical disruption (MCD) via PEI and HIFU inhibits uncontrolled proliferation and reduces tumorigenicity and aggressiveness of liver cancer cells through ROS-mediated activation of TNF and Fas apoptotic pathways. To test this hypothesis, we studied *in vitro* the effects of MCD on liver cancer cell viability, proliferation, tumorigenicity, metastatic potential, ROS production, and expression of death receptors and cancer stem cell markers. We also investigated tumorigenicity post MCD therapy in a xenograft model of liver cancer *in vivo*.

## Materials and Methods

### Cell culture

Non-tumorigenic (HepG2) and tumorigenic (Hep3B, PLC/PRF/5) HCC cell lines were purchased from ATCC (Manassas, VA). Primary human umbilical vein endothelial cells (HUVEC) were procured from Thermo Fisher Scientific (Waltham, MA). HCC cells were cultured in high-glucose DMEM (Thermo Fisher Scientific) containing 10% USDA-origin fetal bovine serum and 1% penicillin-streptomycin (Thermo Fisher Scientific). HUVEC were cultured in Medium 200 supplemented with Low Serum Growth Supplement, 10 µg/ml gentamicin, and 0.25 µg/ml amphotericin B (Thermo Fisher Scientific). Prior to an experiment, the cells were incubated at 37 °C with 5% CO_2_ until reaching 70% confluence.

### High-intensity focused ultrasound (HIFU)

The HIFU signal was applied using a 1.1 MHz, single-element, concave transducer (model H102, Sonic Concepts, Bothell, WA) with stainless steel housing and 64-mm active diameter. The transducer bandwidth was  0.748 to 1.380 MHz. Its  geometric focal length, transverse focal width, and axial focal length were 63.2 mm, 1.5 mm, and 8 mm, respectively. A cone containing degassed water at 37 °C was coupled to the transducer. The input sinusoidal signal was produced by a 33220 A function generator (Agilent Technology, Santa Clara, CA) and passed through a fixed gain (50 dB) ENL 2100 power amplifier (Electronics & Innovation, Rochester, NY) before entering the transducer. A 2 Giga-samples/s InfiniiVision DSO-X-2014A oscilloscope (Agilent) monitored the strength of the HIFU signal. Temperature during HIFU treatment was measured using a mini-hypodermic Copper Constantan type T 200-μm thick bare-wired thermocouple (Omega Engineering, Stamford, CT) connected to a temperature meter (SDL200, Extech Instruments, Waltham, MA). HIFU power levels of H2 (acoustic output power P = 2.7 W, spatial peak temporal average intensity I_SPTA_ = 0.24 kW/cm^2^), H4 (P = 8.7 W, I_SPTA_ = 0.70 kW/cm^2^), and H5 (P = 12 W, I_SPTA_ = 0.88 kW/cm^2^) were used in the study. Samples were exposed to continuous HIFU for 30 seconds.

### *In vitro* treatment procedure

Prior to treatment, 2.7 × 10^6^ HCC cells were resuspended in 100 µL of growth medium in a thin-wall 0.2 mL microcentrifuge tube (Bio-Rad, Hercules, CA). Cells  were then centrifuged at 2000 rpm for 2 minutes, forming a dense cell pellet. The cell pellets were separated into six different treatment groups: 1) Control, 2) 4% ethanol (Ethanol), 3) H4, 4) Ethanol + H4 (E + H4), 5) H5, and 6) Ethanol + H5 (E + H5). Ethanol was added immediately prior to HIFU exposure.

### Viability/apoptosis

After treatment, cancer cells were re-cultured for 2, 24, and 72 h post-treatment. Viability, early apoptotic and late apoptotic/necrotic cell populations were measured using flow cytometry and an Annexin V/PI Apoptosis Detection Kit (Thermo Fisher Scientific). The cells were washed with PBS and then binding buffer. Next, the cells were incubated with 195 μL binding buffer and 5 µL Annexin V at room temperature for 10 minutes and then washed twice with binding buffer. 10 μL of Propidium Iodide (PI, 20 μg/ml) was added to the cell suspension immediately prior to flow cytometry. 100,000 events, excluding aggregates and particulates, were collected in the forward and side-scatter gates using the Attune Acoustic Focusing Cytometer (Applied Biosystems, Grand Island, NY). Apoptotic and necrotic cells were identified by green fluorescence (Annexin V) and red fluorescence (PI), respectively. Cells that stained PI negative and Annexin V positive were considered early apoptotic, while late apoptotic/necrotic cells were both PI and Annexin V positive.

### Proliferation

Cellular proliferation was measured using the WST-8 Cell Proliferation Kit (Caymen Chemical, Ann Arbor, MI). In this experiment, 10^4^ treated cells in 100 μL of medium were placed in each well of a 96-well plate and incubated for 24, 48, and 72 h. 10 μL of a mixture of equal volume WST-8 and Electron Mediator Solution was added to each well and mixed at 150 rpm on an orbital shaker for one minute. Cells were then incubated for two hours and gently mixed again for one minute. Absorbance of each sample was measured at 540 nm using a microplate reader (ELx808, BioTek Instruments, Winooski, VT).

### Long-term culture

Cells were re-cultured in 35 mm petri dishes post-treatment and adherent cells were counted every day for up to 14 days. The growth medium was changed daily and 10 images per sample were taken at 4× magnification for assessment of growth rate and proliferative potential. The average number of cells per image was plotted for different treatment groups and days of culture. If cell confluence was reached, the cell culture was terminated in 2 days.

### ROS expression

A chloromethyl (CM) derivative of H_2_DCFDA (Thermo Fisher Scientific) was utilized to measure ROS expression. The cells were incubated in a culture medium mixed with 100 μM of CM-H2DCFDA for 2 h before treatment and for 24, 48, and 72 h post-treatment. 100 μM hydrogen peroxide (H_2_O_2_) was used as positive control. Note that CM-H_2_DCFDA is particularly sensitive to H_2_O_2_^26,27^. Cold PBS was used to wash the cells before flow cytometric analysis. Each sample was excited at 495 nm, and emission was observed at 520 nm.

### Membrane protein expression

Mouse anti-human antibodies to membrane proteins TNFR1 (H398), Fas (DX2), CD49f (GoH3), CD90 (5E10), and CD133 (EMK08) were purchased from Thermo Fisher Scientific. HCC cells were washed with PBS and then with fluorescence-activated cell sorting buffer, composed of 2% BSA and 0.1% sodium azide in PBS. FITC-conjugates mouse IgG and mouse anti-human antibodies for the protein were added to the washed cells. The cells and antibodies were then incubated on ice for 45 minutes, after which they were washed by the buffer and resuspended in the buffer with 2% formaldehyde. The cells were analyzed via flow cytometry at 2, 24, and 72 h post-treatment.

### Death receptor blocking assay

HCC cells were incubated with 10 µg/mL mouse anti-human TNFR1 monoclonal antibody (H398, Thermo Fisher Scientific) and 10 µg/mL mouse anti-human Fas monoclonal antibody (ANT-205, Prospec-Tany TechnoGene, Rehovot, Israel) at 37 °C for 2 h prior to treatment to block death receptors TNFR1 and Fas. Treated HCC cells were re-cultured in 12 well plates with 2 µg/mL TNFR1 antibody and 2 µg/mL Fas antibody. Viable, early apoptotic, and late apoptotic/necrotic populations of cancer cells were determined at 24 and 72 h post-treatment by flow cytometry using an Annexin V-FITC Apoptosis Detection Kit (Thermo Fisher Scientific).

### ROS inhibition assay

ROS expression in cancer cells was inhibited by NAC (N-acetyl-L-cysteine) and BHA (Butylated hydroxyanisole), purchased from Sigma-Aldrich. Specifically, the cells were incubated in growth medium containing 1 mM NAC and 10 uM BHA for 30 min and then treated by ethanol and/or HIFU. Immediately after treatment, the cells were re-cultured for 24 and 72 h in the medium with 500 µM NAC and 5 μM BHA.

### Colony-forming unit assay

Treated HCC cells were counted and seeded into 60 mm × 15 mm petri dishes at 500 cells/dish. Cells were incubated for 14 days, after which the growth medium was removed, and each dish was washed with PBS. The colonies were then fixed with formalin for 30 minutes and then stained with 0.2% methylene blue for 30 minutes. Excess methylene blue was washed off with deionized water. Dishes were imaged, and cell colonies were counted using ImageJ software (NIH, Bethesda, MA).

### Scratch-wound assay

HCC cells were seeded into 12-well plates and grown until confluence. Upon confluence, a 200 μL pipet tip was steadily scraped across the surface of the single cell monolayer. To create scratches of similar width, the pipet tip was held directly perpendicular to the cell surface throughout the scratch. Images of the scratched region were taken daily until complete closure of the wound. ImageJ was used to measure scratch width at each timepoint. In this experiment, cells were exposed to sublethal HIFU power (H2) to ensure that they were viable enough to form a confluent monolayer.

### Static adhesion

HUVEC were seeded in a 96-well plate at 0.3 × 10^6^ cells/mL after passage 3 or 4 and cultured overnight to reach confluence. HUVEC were activated by incubating with 10 ng/mL TNF-α (Sigma Aldrich) for 4 h. The HUVEC growth medium was then replaced with DMEM containing 2.0 × 10^4^ viable DiO-labeled HCC cells. The HCC cells were allowed to settle and form adhesive contacts with HUVEC for 15 minutes. The DMEM solution was then removed, and each well was washed with PBS three times, eliminating free-floating cancer cells. Adherent cells were visualized with an inverted epi-fluorescent microscope (Nikon Eclipse Ti-S, Tokyo, Japan) with a 10× objective. Images were captured using a digital CCD camera (Qimaging Retiga EXi, Surrey, Canada) at five different locations in each well. The number of adherent cells was determined with a custom image processing MATLAB code (Mathworks, Natick, MA). The image field size was 904 µm × 675 µm. Static adhesion tests were conducted at 2, 24, and 72 h post-treatment.

### Spheroid formation

The hanging drop method was utilized to grow HCC tumor spheroids. Treated cells were seeded in a hanging-drop plate (3D Biomatrix, Ann Arbor, MI) at a concentration of 10^3^ cells/μL in growth medium. Images of drops were taken at 24 and 72 h post-treatment. Tumor spheroid formation was blindly scored on a 0–2 scale, with a 0 being no spheroid formation, a 1 being formation of a loose spheroid, and a 2 being dense spheroid formation.

### Animal study

All procedures using animals were approved by Tulane University’s Institutional Animal Care and Use Committee (Protocol 0440 R). They were carried out under guidelines of the Association for Assessment and Accreditation of Laboratory Animal Care (AAALAC).

### *In vivo* tumor xenograft model

The four-week-old male and female Nu/Nu athymic nude mice were purchased from Jackson labs (J:NU 007850, Bar Harbor, ME) and allowed one week of acclimation. A 200 μL bolus containing 1.0 × 10^6^ Hep3B cells in PBS and Matrigel Matrix High Concentration (Corning, Corning, NY) mixed in a 1:1 volume was subcutaneously injected on the left and right flanks of each animal via a 28-gauge needle. Digital calipers and a SonoSite 180+ ultrasound imaging system (SonoSite, Bothell, WA) were used to measure tumor dimensions. The tumor volume was calculated using the following formula: *V* = *π*/6 × 2*a* × *b*, where *a* is the short axis and *b* the long axis of the tumor. Once the tumor volume reached approximately 200 mm^3^, mice were randomly assigned to a specific treatment group. There was a total of 10 tumors per group.

### *In vivo* treatment

Prior to treatment, Isoflurane gas (Vet One, Meridian, ID) was used to anesthetize mice. Mice were held in a custom-made restrainer, allowing access of tumor site for treatment and imaging. The sham group was injected with 50 μL of PBS, while mice in ethanol treatment groups were injected with 50 µL of 99% ethanol (Sigma Aldrich). A 3-D positioning system (Thorlabs, Newton, NJ) and diagnostic ultrasound aligned the focus of the HIFU signal to the tumor. Ultrasound gel (Aqua Sonic 100, Parker labs, Fairfield, NJ) applied between the cone and skin served as the coupling media. With the HIFU beam at power level H5, the 8–10 mm diameter tumors were ablated with three to four HIFU shots at 30 second intervals. Ethanol was injected immediately prior to HIFU exposure in the E + H5 treatment group. The tumor volume was measured daily for 14 days post-treatment. No additional treatment was administered. It should be noted that the injection of 50uL ethanol leads to the intratumoral ethanol concentration between 11% (for 1 cm tumors) and 20% (for 0.8 cm tumors), which is expected to reduce tumor cell viability (cf. Fig. S1 in ref. ^28^). This dose, however, is much less than the PEI clinical dose of 5 to 60 ml per 1 to 3 cm tumor, which would lead to fatal intoxication in mice. As tested in the prostate cancer study^28^ and current work, the injection of 50 μL ethanol does not induce animal death and, in fact, it does not change the animal survival rate as compared to tumor-bearing animals in the control group.

### Histological analysis

Tumor tissue collection occurred at either day 5 or day 14, when mice were sacrificed via CO_2_ asphyxiation. Collected tissue specimens were fixed in formalin for 24 h before being embedded into paraffin. Embedded tissues were partitioned into 4μm thick slices before being placed onto glass sides and stained with Hematoxylin and Eosin (H&E). The study pathologist blindly evaluated the control and treatment slides for maximal tumor diameter and % necrosis.

### Statistical analysis

Results were evaluated with an unpaired *t*-test by using GraphPad Prism 8.1 (GraphPad Software, La Jolla, CA, USA). Statistically significant differences, set to *p* < 0.05 between experimental groups, were measured by the multiple comparison Holm-Sidak method. The statistical data are represented as mean ± standard error of the mean (SEM). The number of independent tests is listed in figure legends.

## Results

### MCD reduces viability and proliferation of human liver cancer cells

**Figure 1:**
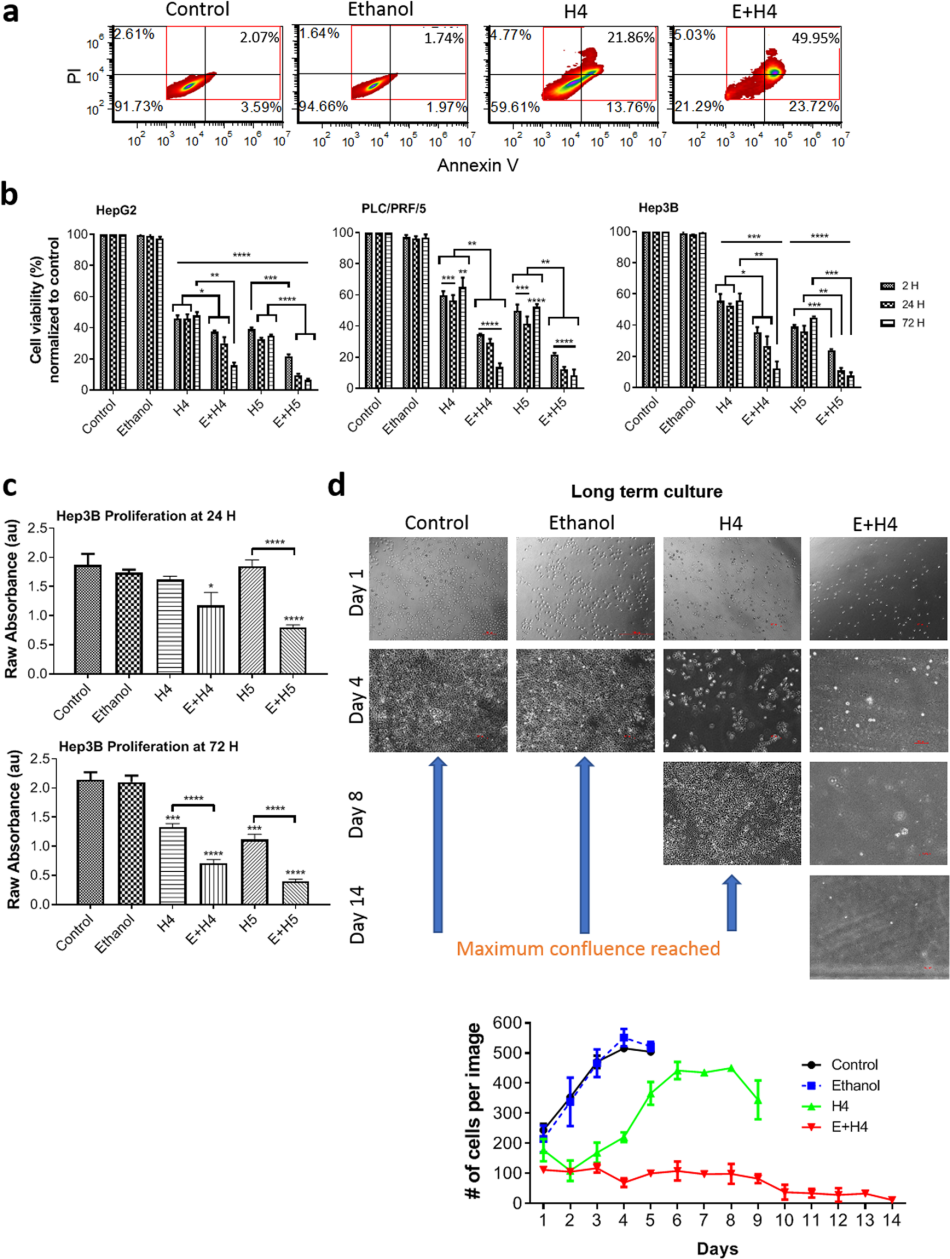
Reduction of viability and proliferation of HCC cells upon exposure to mechanochemical disruption (E + H). (**a**) Representative density plots of viable, apoptotic, and necrotic Hep3B cells at 72 h post-treatment, obtained by Annexin V/Propidium Iodide (PI) flow cytometric assay. Viable and early apoptotic cells are in the lower two quadrants of the plot (stained PI negative), while the necrotic/late apoptotic cells are in the upper two quadrants (PI positive). Apoptotic cells are Annexin V positive (the right quadrants). (**b**) Viability of HepG2, PLC/PRF/5, and Hep3B cells, defined as population percentage of late apoptotic and necrotic cells normalized to control, at 2, 24, and 72 h post-treatment with ethanol, HIFU levels 4 and 5 (H4, H5), and a combination of ethanol and HIFU (E + H4, E + H5). (**c**) Hep3B cell proliferation was measured by the WST-8 assay at 24 and 72 h post-treatment. (**d**) Representative images (left) and growth rate (right) of Hep3B cells at different days (1–14) of post-treatment culture. Values are mean ± SEM of 3 (**b**,**d**) or 5–9 (**c**) independent experiments. *p < 0.05, **p < 0.01, ***p < 0.001, ****p < 0.0001. Asterisks without brackets refer to differences between treatment and control groups. Asterisks with brackets are used for comparison among treatment groups.

Flow cytometry for Annexin V and PI was performed on HepG2, PLC/PRF/5, and Hep3B liver cancer cells (Fig. 1a,b). The representative flow cytometry maps in Fig. 1a show the distribution of viable, early apoptotic, late apoptotic, and necrotic Hep3B cells left untreated (control) or treated with 4% ethanol (Ethanol), HIFU level H4 alone, or a combination of Ethanol and HIFU level H4 (E + H4) at 72 h. Using the percentage of viable cells from these maps, the cellular viability normalized to control was plotted in Fig. 1b for different cell lines, experimental groups, and three time instances (2, 24, and 72 h post-treatment). As seen in Fig. 1b, treatment with ethanol alone did not significantly decrease cellular viability in any cell line at any time point (>96% of the control group viability). Cells treated with HIFU alone had significantly lower viability than cells in the control or ethanol alone groups at all time points (p < 0.01). Standalone HIFU at level H4 decreased cellular viability to 48.0 ± 2.1% for HepG2, 65.2 ± 5.9% for PLC/PRF/5, and 55.9 ± 4.5% for Hep3B cell lines at 72 h. However, a further increase in HIFU power only modestly reduced cell survival (11–13% drop in cell viability between H4 and H5). The pre-exposure of cancer cells to 4% ethanol significantly (*p* < 0.01) reduced viability of H4-treated cells to 15.8 ± 1.9% for HepG2, 13.6 ± 2.5% for PLC/PRF/5, and 12.1 ± 4.6% for Hep3B lines at 72 h. Less than 9% of cells remained viable post ethanol + H5 treatment at this time point. The data in Fig. 1b also demonstrated that cellular viability in the combination treatment group continuously decreased with time, while cells post standalone HIFU had no significant temporal difference in viability.

Changes in proliferation of Hep3B cells (Fig. 1c) and HepG2 and PLC/PRF/5 cells (Supplementary Fig. S1) post-treatment were determined by a WST-8 proliferation assay. The proliferative potential of cells treated with a combination of ethanol and HIFU was significantly (*p* < 0.05 for all E + H groups reaching the value of < 0.001 for Hep3B and HepG2 cells in the E + H5 group) lower than that of control cells early on (24 h). The difference in proliferation between the combination treatment and control groups became even larger with time (72 h). Ethanol alone treatment had no effect on proliferation of liver cancer cells. The combination treatment significantly reduced the proliferation rate at 72 h, as compared to standalone HIFU treatment (*p* < 0.05 for H5 vs E + H5). To confirm that the combination treatment has a long-term effect on cell proliferation, we cultured treated cells for up to 14 days. As illustrated in Fig. 1d, control and ethanol-treated cells reached confluence at day 4 post-treatment. Cells exposed to standalone HIFU took 8 days to reach this stage. The cells treated with both ethanol and HIFU grew very slowly and were unable to form a confluent monolayer even by 14 days.

### MCD causes ROS overproduction and apoptosis via Fas and TNFR1

**Figure 2:**
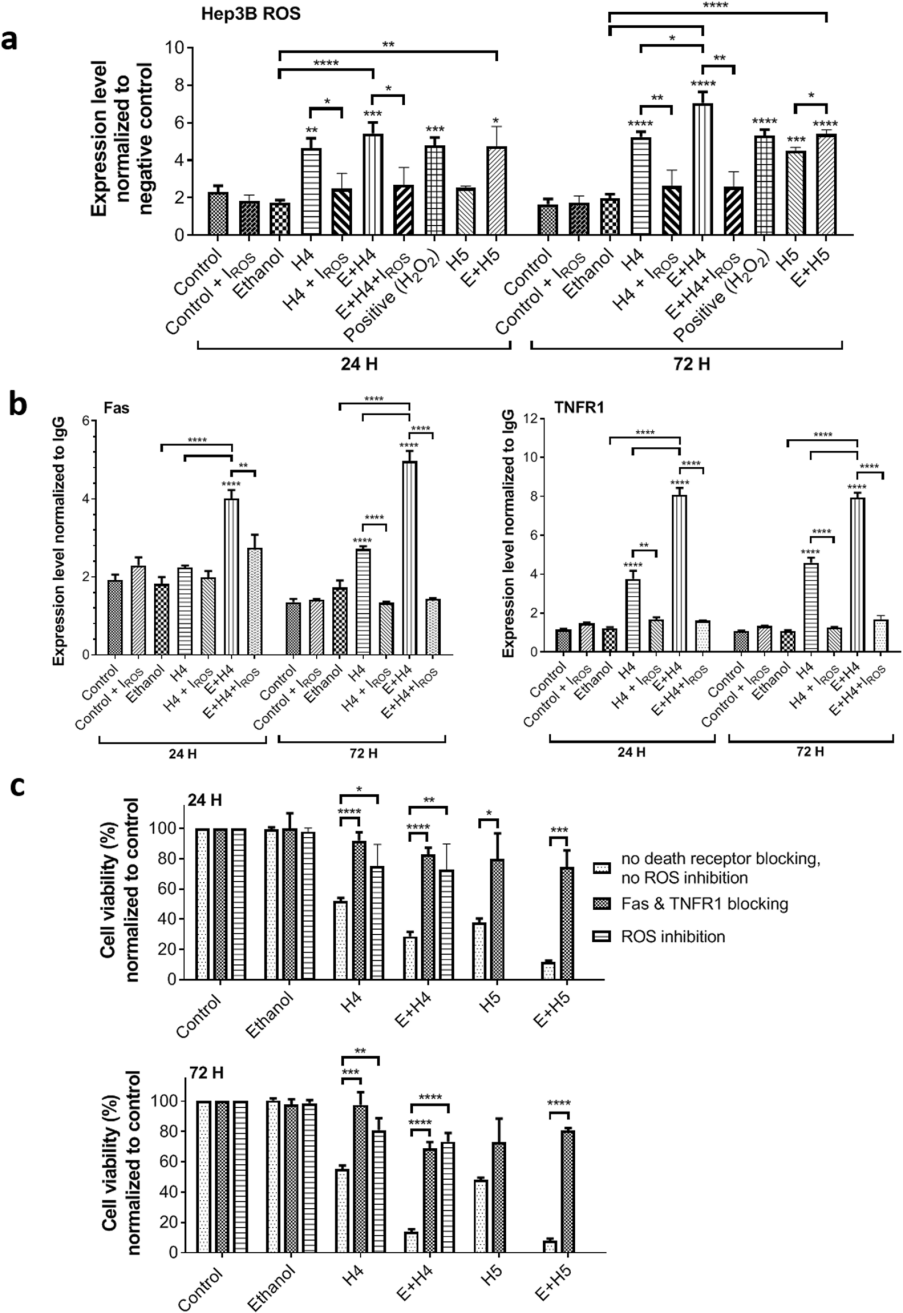
Overproduction of ROS and increased expression and activity of TNFR1 and Fas in Hep3B cells after mechanochemical disruption. (**a**) ROS production in treated cells exposed or not to ROS inhibitors NAC and BHA (I_ROS_), measured by CM-H2DCFDA flow cytometry. (**b**) Expression level of death receptors Fas and TNFR1, relative to isotype control, in treated cells exposed or not to ROS inhibitors. (**c**) Changes in viability of treated cells due to blocking Fas and TNFR1 activity or ROS inhibition, as measured by Annexin V/PI flow cytometry. Shown are the data collected at 24 and 72 h post-treatment. Values are mean ± SEM of 4–8 (**a**), 4–7 (**b**), or 3–9 (**c**) independent experiments. *p < 0.05, **p < 0.01, ***p < 0.001, ****p < 0.0001. Asterisks without brackets refer to differences between treatment and control groups. Asterisks with brackets are used for comparison among treatment groups.

ROS production in Hep3B cells significantly increased (*p* < 0.01 at 24 h, and *p* < 0.0001 at 72 h) post-treatment with standalone HIFU at level H4 or its combination with 4% ethanol (4.65 ± 0.53 for H4 vs 2.30 ± 0.35 for no treatment at 24 h; 5.25 ± 0.28 for H4 vs 1.66 ± 0.28 for no treatment at 72 h), as evident in Fig. 2a. The level of ROS in the combination treatment groups was higher than that of individual treatment groups and even exceeded the ROS level in cells exposed to 100 μM H_2_O_2_ (positive control) at 24 h (5.44 ± 0.59 for E + H4 and 4.76 ± 1.04 for E + H5 vs 4.78 ± 0.44 for positive control) and 72 h (7.06 ± 0.59 for E + H4 and 5.39 ± 0.23 for E + H5 vs 5.32 ± 0.32 for positive control). The ROS level in untreated Hep3B cells was 2.30 ± 0.35 at 24 h and 1.66 ± 0.28 at 72 h. HepG2 and PLC/PRF/5 cells also showed significant overproduction of ROS post combination treatment (Supplementary Fig. S2). By incubating the cells with NAC and BHA (ROS inhibitors, I_ROS_), the ROS expression level in H4- or E + H4-treated cells reduced to less than 2.7, which was insignificantly different from the ROS level in untreated cells.

The expression and activity of death receptors Fas and TNFR1 are dependent on ROS^29,30^. Figure 2b and Supplementary Fig. S3 point to a significant correlation between ROS production and expression of Fas and TNFR1 in treated liver cancer cells. In particular, only the combination treatment group, where ROS was overproduced, had a significantly higher Fas expression in Hep3B cells than the control group at both 24 h (4.02 ± 0.21 for E + H4 vs 1.92 ± 0.14 for control, ~2.1 times higher, *p* < 0.0001) and 72 h (4.97 ± 0.26 for E + H4 vs 1.35 ± 0.08 for control, ~3.7 times higher, *p* < 0.0001). TNFR1 expression significantly increased in all HIFU-treated Hep3B cells at both 24 and 72 h (*p* < 0.0001) as compared to control, and there was also a highly significant difference in the expression between standalone HIFU and ethanol + HIFU treatment groups (*p* < 0.0001). The level of TNFR1 expression was 8.09 ± 0.94 in the E + H4 group at 24 h and 7.95 ± 0.63 at 72 h, ~7 times higher than that in the control group (1.15 ± 0.13 at 24 h and 1.07 ± 0.10 at 72 h). The HIFU or ethanol + HIFU treated Hep3B cells incubated with ROS inhibitors did not express Fas and TNFR1 at the level significantly higher than the control cells (I_ROS_ groups in Fig. 2b). For the combination treatment, ROS inhibition reduced Fas expression to 2.75 ± 0.33 at 24 h (1.46 times less, *p* < 0.01) and 1.44 ± 0.02 at 72 h (3.45 times less, *p* < 0.0001). A much larger effect of ROS inhibition was seen on TNFR1 expression. In the combination treatment group, the TNFR1 level dropped to 1.61 ± 0.08 at 24 h (5.02 times less, *p* < 0.0001) and 1.69 ± 0.36 at 72 h (4.70 times less, p < 0.0001) post incubation with ROS inhibitors. ROS inhibition also led to a significant decrease (*p* < 0.01) in TNFR1 expression in the cells treated by standalone HIFU. These data indicate that ROS has a direct effect on the upregulation of death receptor expression in the cells undergoing mechanical disruption.

When both Fas and TNFR1 were blocked, the viability of treated Hep3B cells was recovered to 66% - 95% of the unblocked control group and no significant differences in viability were seen between treated and untreated cells after blocking (Fig. 2c). In the E + H4 group, death receptor blocking increased cell viability from 26.7 ± 6.1% to 77.9 ± 5.5% at 24 h and from 13.9 ± 4.9% to 68.8 ± 7.4% at 72 h (>51% increase in viability due to blocking). In the E + H5 group, blocking led to an increase in cell viability from 10.9 ± 1.6% to 69.6 ± 9.5% at 24 h and from 7.9 ± 3.6% to 80.6 ± 3.1% at 72 h (>59% increase). A significant increase in cell viability was also seen after inhibiting ROS in the cells treated by standalone HIFU (*p* < 0.05 at 24 h, *p* < 0.01 at 72 h) or ethanol + HIFU (*p* < 0.01 at 24 h, *p* < 0.0001 at 72 h). In particular, in the E + H4 group, the cell viability increased to 72.2 ± 17.8% of untreated cells at 24 h (46% increase) and 73.3 ± 9.9% at 72 h (60% increase). The viability of the cells where ROS was inhibited was not significantly different from the cell viability where death receptors were blocked. These data point out that ROS has a direct cause-effect relationship with Fas and TNFR1 death receptor activity. Overall, the results described in this section confirm that ROS overproduction induced by mechanochemical disruption causes cell death via Fas and TNFR1.

### MCD diminishes the metastatic potential of liver cancer cells

**Figure 3:**
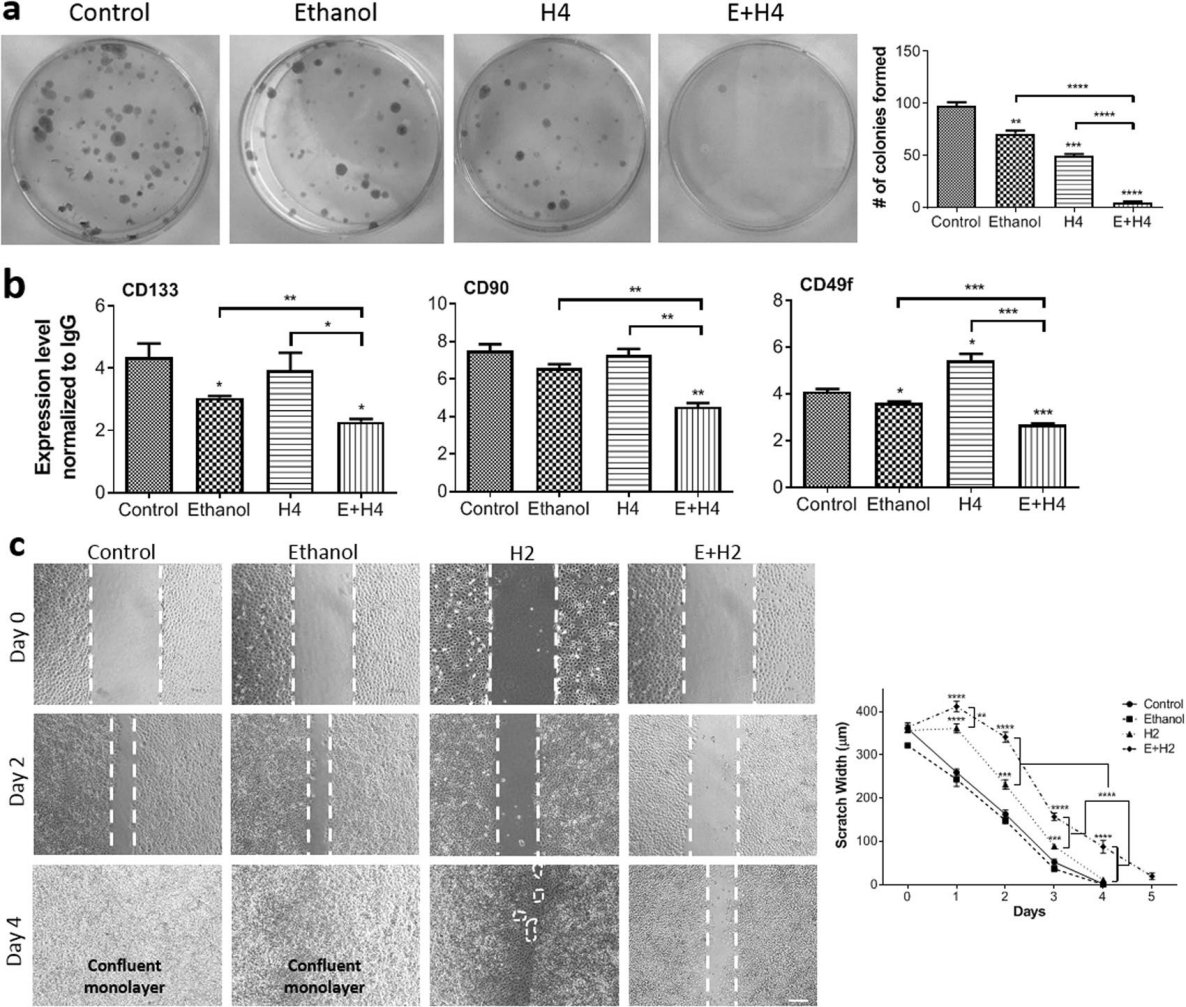
Decreased stemness and migratory ability of Hep3B cells after mechanochemical disruption. Cell stemness post-treatment was assessed by the colony forming unit (CFU) assay and the expression of liver cancer stem cell markers. (**a**) Representative images of colonies after staining with methylene blue (left) and average number of colonies formed per treatment group (right). (**b**) Normalized-to-IgG expression of CD133 (Prominin 1, left), CD90 (Thy1, middle), and CD49f (α_6_ integrin, right) on treated cells at 72 h post-treatment. (**c**) The migratory ability of cells treated with ethanol and/or sublethal HIFU 2 (H2) assessed by a scratch-wound assay. Shown are representative images of wounds for different treatment groups (left) and scratch width (right) at multiple time points. Dashed white lines depict borders of acellular regions. Values are mean ± SEM of 3 (**a**,**b**) or 6–18 (**c**) independent experiments. *p < 0.05, **p < 0.01, ***p < 0.001, ****p < 0.0001. Asterisks without brackets refer to differences between treatment and control groups. Asterisks with brackets are used for comparison among treatment groups.

The presence of cells with stem-like properties within a liver tumor is indicative of highly aggressive, metastatic liver cancer^31^. The colony-forming unit (CFU) assay can measure the stemness of cancer cells and their ability to regrow the tumor post-treatment^32^. According to the data in Fig. 3a, treatment of Hep3B cells with a combination of ethanol and HIFU nearly abolished their colony-forming ability (~20 fold decrease in stemness). With a seeding density of 500 viable cells, control Hep3B cells formed 98.0 ± 3.2 colonies in 14 days. The number of colonies reduced to 71.0 ± 5.3 for ethanol-treated cells (*p* < 0.01), 49.7 ± 3.1 for cells treated with standalone HIFU (H4, *p* < 0.001), and 4.7 ± 2.1 for E + H4-treated cells (*p* < 0.0001). Statistically significant differences (*p* < 0.0001) in the number of colonies were seen between the combination treatment and individual treatment groups. The stemness of HepG2 and PLC/PRF/5 cells also significantly (p < 0.01) reduced with the combination treatment, as compared to untreated cells or cells exposed to ethanol alone or HIFU alone (Supplementary Fig. S4a). The decrease in cancer cell stemness post combination treatment was confirmed by analysis of liver cancer stem cell (CSC) markers CD133, CD90, and CD49f. In particular, the expression of these markers significantly reduced (*p* < 0.05 at least) in Hep3B cells (Fig. 3b) and HepG2 and PLC/PRF/5 cells (Supplementary Fig. S4b) post E + H4 treatment.

It has been established that the expression of CSC markers in carcinomas including HCC is associated with epithelial-to-mesenchymal transition by which tumor cells acquire a highly invasive, migratory phenotype^33^. The scratch-wound assay data in Fig. 3c indicates that untreated and ethanol-treated Hep3B cells are highly migratory, closing a gap of 360.5 ± 9.4 μm within 3 days. The cells in the HIFU level 2 (H2) and combined E + H2 groups required one and two more days to close such a gap, respectively. The H2 treatment group had a significantly wider gap than the control group at day 1 (*p* < 0.0001) and day 3 (*p* < 0.001). The gap in the E + H2 group was significantly wider (*p* < 0.0001) than all other treatment groups at day 1, 2, 3, and 4. Thus, Hep3B cells exposed to combination treatment have a diminished migratory ability.

**Figure 4:**
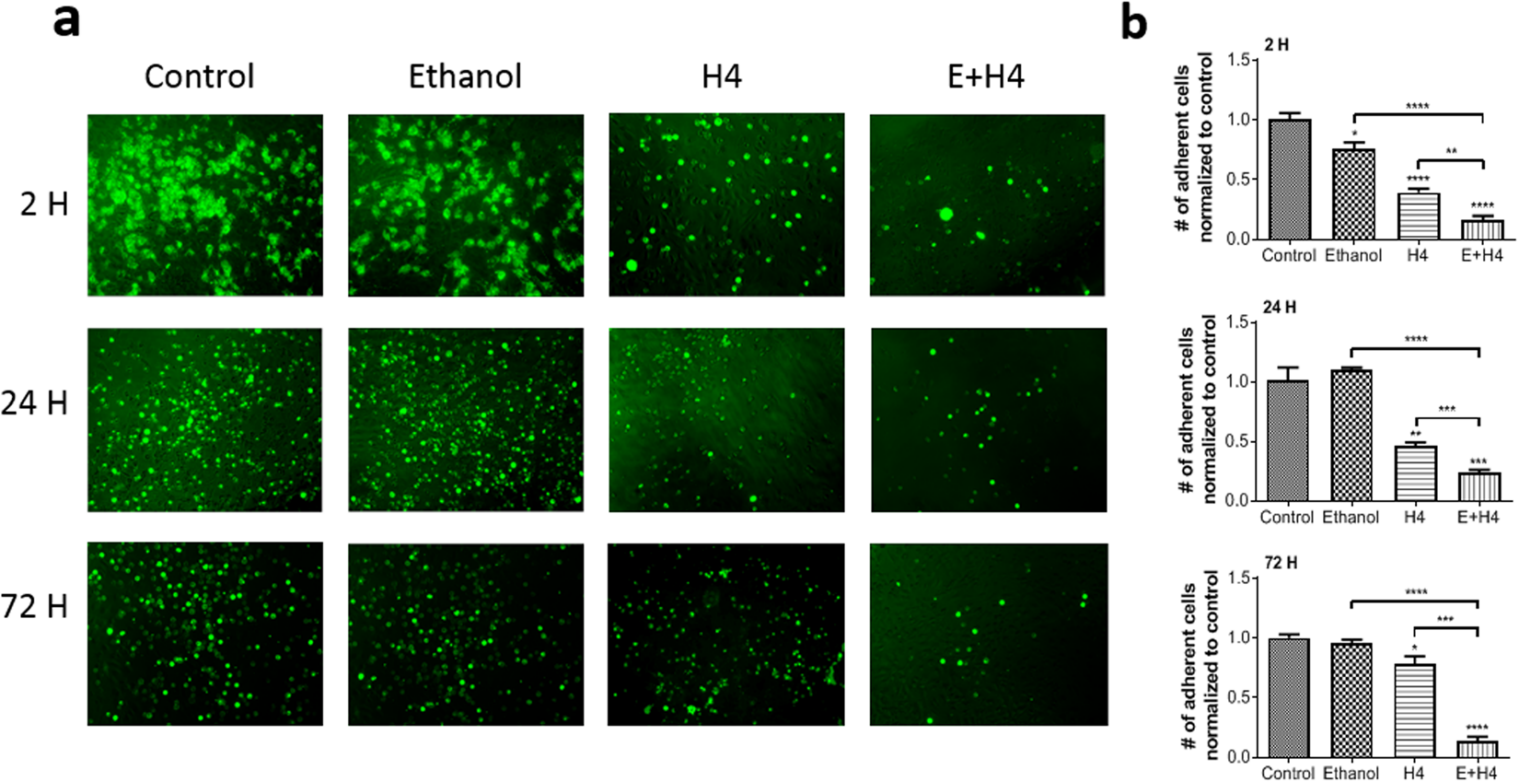
Mechanochemical disruption weakens adhesion of Hep3B cells to vascular endothelium. (**a**) Representative images of adherent DiO-labeled cells (green) from various treatment groups on TNF-α- activated HUVEC. (**b**) Number of adherent cancer cells normalized to the control. Values are mean ± SEM of 4 independent experiments. *p < 0.05, **p < 0.01, ***p < 0.001, ****p < 0.0001. Asterisks without brackets refer to differences between treatment and control groups. Asterisks with brackets are used for comparison among treatment groups.

Highly invasive cancer cells break away from the primary tumor and spread via the blood and lymphatic circulation throughout the body. To form micrometastases at distant sites, circulating cancer cells must adhere and then migrate through local endothelium. Thus, cancer cell adhesion to endothelium is critically important for secondary tumor formation. Figure 4a shows representative images of treated DiO-labeled Hep3B cells attached to TNF-α activated vascular endothelium (HUVEC). These images reveal a strong and lasting reduction in Hep3B cell adhesion after MCD. Adherent cells were counted at 2, 24, and 72 h post-treatment and normalized to the control (Fig. 4b). Hep3B cells treated with ethanol alone had no significant difference with respect to the control at 24 and 72 h. The E + H4 treatment group maintained the most significant decrease in cell adhesion with respect to the control at all time points (*p* < 0.001), while other experimental groups regained most of the cell adhesion potential by 72 h. Cell adhesion in the E + H4 group was significantly lower than that in the ethanol group or the H4 group (*p* < 0.0001 for ethanol vs. E + H4; *p* < 0.01 at 2 h and *p < *0.001 at 24 and 72 h for H4 vs E + H4). Supplemental Fig. S4c also demonstrate reduction in the adhesion potential in HepG2 and PLC/PRF/5 cells post the combination treatment. The observed reduction in stemness as well as migratory and adhesive potentials indicates that HCC cells post MCD have a diminished metastatic phenotype.

### MCD reduces tumorigenicity of liver cancer *in vitro* and *in vivo*

**Figure 5:**
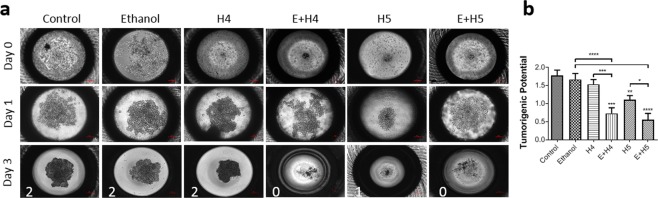
Mechanochemical disruption reduces Hep3B tumorigenicity *in vitro*. (**a**) Representative images of tumor spheroids from treated Hep3B cells at day 0, 1, and 3 of hanging-drop culture. (**b**) Tumorigenic potential of the cells at day 3 post treatment, assessed based on a 2-point scale, with 0 being no spheroid formation, 1 being loose spheroid formation, and 2 being dense spheroid formation. Values are mean ± SEM of 9–15 independent experiments. *p < 0.05, **p < 0.01, ***p < 0.001, ****p < 0.0001. Asterisks without brackets refer to differences between treatment and control groups. Asterisks with brackets are used for comparison among treatment groups.

The observed decrease in stemness of treated Hep3B cells indicates that their ability to form tumors may be compromised. Figure 5 confirms that Hep3B cells exposed to both ethanol and HIFU lose this ability. As seen in Fig. 5a, densely packed tumor spheroids were formed in control, ethanol alone, and H4 alone treatment groups by day 3 of culture, indicating strong tumorigenicity. Increasing the power of standalone HIFU to H5, slightly but significantly (*p* < 0.01) reduced tumorigenicity, as evident by less dense spheroid formation. Both E + H4 and E + H5 groups showed no or loose spheroid formation. The tumorigenic potential of liver cancer cells, quantified based on blind scoring (0 = no spheroid, 1 = loose spheroid, 2 = dense spheroid), is shown in Fig. 5b. The cells in the combination treatment groups had significantly lower tumorigenic scores than control (*p* < 0.001), ethanol alone (*p* < 0.0001), or HIFU alone groups (*p* < 0.05). A significant reduction in tumorigenicity was also seen in HepG2 and PLC/PRF/5 cells (Supplementary Fig. S5) exposed to a combination of ethanol and HIFU, as compared to untreated cells (*p* < 0.001) or treated with ethanol alone (*p* < 0.001) or HIFU alone (*p* < 0.05).

**Figure 6:**
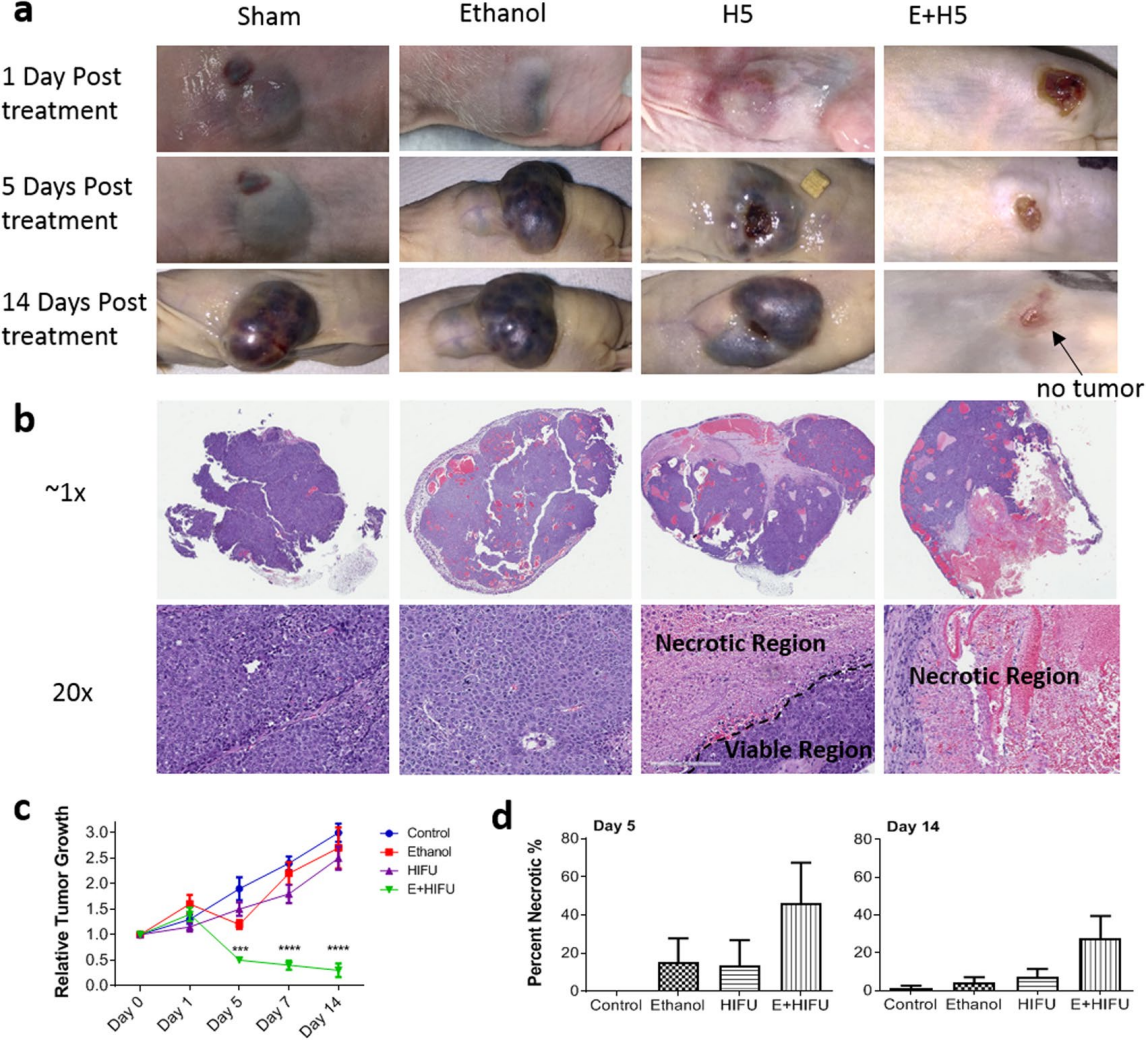
Mechanochemical disruption leads to tumor regression *in vivo*. (**a**) Representative images of Hep3B xenografted tumors at day 1, 5, and 14 post treatment. (**b**) Images of H&E stained tumor tissues collected at day 14 post-treatment, at 1× and 20× magnification. (**c**) Change in tumor volume, normalized to its pre-treated value, with post-treatment time. (**d**) Percent necrosis (%) of tumors collected at day 5 (left) and 14 (right). Values are mean ± SEM of 5 (**c**) or 3–4 (**d**) independent experiments. ****p < 0.0001.

The presented *in vitro* data in Fig. 5 and Supplementary Fig. S5 indicate that MCD makes liver cancer cells less capable of tumor formation. To test whether this disruptive therapy can stop tumor progression and potentially lead to tumor regression *in vivo*, we utilized a Hep3B xenograft mouse model. Figure 6a illustrates tumor regression to a no-caliper measurable size in mice treated with both ethanol and HIFU. Tumors continued to grow in all other treatment groups. Images of H&E stained tumor sections in Fig. 6b shows little to no necrosis in sham and ethanol treated tumors, small necrotic regions after standalone HIFU (H5), and large necrotic regions in the E + H5 treated tumors at 14 days post-treatment. The data on tumor growth (Fig. 6c) show that the average tumor size was reduced to 30% (SD = 30%) of its original size, with many tumors completely regressed, by 14 days post E + H5 treatment. During this time period, tumors in the sham, ethanol alone, and H5 alone groups grew to at least 250% (SD = 50%) of the original size. Starting from day 5 post-treatment, there was a statistically significant difference (*p* < 0.0001) between the sham and E + H5 groups.

Blind pathological evaluation of resected tumors revealed that the combination treatment resulted in a greater % necrosis at 5 and 14 days than other treatment groups (Fig. 6d). At 5 days, E + H5 histological slides showed 46.25 ± 21.15% necrosis, as opposed to no necrosis for the sham group. In comparison, ethanol alone and H5 alone tumors at 5 days exhibited 15.3 ± 12.4% and 13.7 ± 13.2% necrosis, respectively. The disparity between the E + H5 and other treatment groups increased even more by 14 days post-treatment. The E + H5 tumors were 27.8 ± 11.8% necrotic, while the sham, ethanol alone and H5 alone tumors were 1.3 ± 1.3%, 4.0 ± 3.0%, and 7.3 ± 4.1% necrotic, respectively.

## Discussion

HCC is a highly fatal cancer with a median survival time of 3.6 months if left untreated^34^, which increases only to 11 months if both treated and untreated patients are included^35^. The considerable mortality rate associated with HCC emanates directly from the limited treatment options which are ineffective for the intermediate and advanced stages of this disease. As we showed previously^16,36^, MCD of tumor tissue by a combination of PEI and HIFU increases the tissue ablation volume, reduces acoustic intensity, and decreases number of HIFU shots per tumor. It also decreases the aggressiveness of prostate cancer cells^28^ and, as demonstrated in this work, HCC cells. In particular, MCD therapy prevents uncontrolled proliferation of HCC cells, sensitize the cells to apoptosis, and lowers their metastatic and tumorigenic potentials *in vitro* and *in vivo*.

The local deposition of acoustic energy to a tumor by HIFU causes strong molecular vibrations, leading to the mechanical disruption of cancer cells and tumor microenvironment. When HIFU is combined with ethanol, the cells experience MCD that leads to three possible outcomes: (1) immediate cell death, (2) programmed cell death, (3) cell survival accompanied by phenotypic alterations. The immediate cell death occurs in a subset of cells due to unbearable mechanical, thermal, and oxidative stresses. For example, rapid heating due to acoustic energy absorption coupled with ethanol-induced dehydration and oxidative shock lead to protein denaturation and breakdown, and thus coagulative necrosis of the cells^37,38^. Furthermore, rupture of the cytoskeleton and plasma membrane by extensive mechanical vibrations results in cell lysis^17,39^.

The results of our study suggest that MCD induces ROS overproduction (particularly, overproduction of hydrogen peroxide) that in turn causes programmed death of liver cancer cells via death receptors Fas and TNFR1 (Fig. 2). The role of these receptors in both apoptosis and necroptosis of hepatocytes has been previously demonstrated^40,41^. The intracellular metabolism of ethanol leads to ROS formation in the endoplasmic reticulum (ER)^42^. Similarly, ROS is produced in the cytoplasm as a result of HIFU-induced mechanical disruption^21^. The increase in cytoplasmic ROS due to ethanol and HIFU permeabilizes the mitochondrial membrane, leading to mitochondrial dysfunction and burst release of mitochondrial ROS as well as cytochrome C, apoptosis inducing factor (AIF), and other apoptogenic factors into the cytoplasm^43,44^. This process provides the necessary components for apoptotic cell death and, through the overproduction of ROS, it elevates death receptor activity to ensure the completion of the apoptotic signaling cascade. In a similar fashion, mitochondrial dysfunction and ROS overproduction contribute to TNFR1- and Fas-mediated necroptosis^45^.

Since the liver is a highly vascular organ, favorable conditions exist for metastatic dissemination of liver tumor cells. Even with the best treatment available, there is a high risk that some of the cells survive treatment and progress to a more aggressive phenotype. Ideally, curative treatment should also target surviving cells, e.g., by making them less potent for metastatic transformation and spread. Figures 1, 3 and 4 demonstrate this goal can potentially be achieved by MCD of tumor cells. The cells that experienced this disruption had: (1) very low and even negative proliferation rate in the long run, (2) reduced stemness, both in terms of colony-forming ability and CSC marker expression, and (3) reduced ability to migrate and adhere to vascular endothelium. Surviving cells are also incapable of forming tumors *in vitro* (Fig. 5) and *in vivo* (Fig. 6). These cell reprograming features of MCD therapy maybe associated with inhibition of TNFR1-mediated proliferation and changes in mitochondrial metabolism. In addition to its role as a death receptor, TNFR1 also initiates cell proliferation through NF-κB signaling. Despite an eightfold increase in the TNFR1 expression level, liver cancer cells that survived MCD do not proliferate, suggesting that the NF-κB signaling pathway is impaired. We recently demonstrated that MCD leads to a loss of NF-κB p65 subunit in prostate cancer cells^28^. Also, the overproduction of ROS has been shown to inhibit metabolites in the NF-kB signaling pathway^46^. Mitochondrial permeabilization and destruction induced by MCD either drive the cell to undergo programmed cell death or dramatically reduce the number of viable mitochondria in surviving cells, as indirectly confirmed by WST-8 assay data (Fig. 1c). Recent studies link mitochondrial biogenesis and metabolism with aggressive cancer phenotypes, enhanced tumor growth, and metastasis^47,48^. By depleting mitochondria, MCD therapy makes cancer cells unable to sustain their aggressive behavior.

Overall, the reported *in vitro* and *in vivo* data demonstrate that the mechanical disruption of liver cancer cells by focused ultrasound decreases their tumorigenic and metastatic potentials in the presence of ROS inducing chemical agents, such as ethanol. This MCD approach can potentially be curative for intermediate and advanced stage liver cancer. Further *in vivo* studies with orthotopic and liver metastasis models are required to assess the MCD ability to reduce a risk of tumor recurrence and secondary tumor formation at distant sites.

## Supplementary information

Supplementary Figures
