# Supplementary material for: Phenotypic alterations in liver cancer cells induced by mechanochemical disruption
Source: Sci Rep. 2019 Dec 20;9:19538. doi: 10.1038/s41598-019-55920-2 (PMC6925139; doi:10.1038/s41598-019-55920-2)
Supplement: Supplementary file 1 — Supplementary Figures [file 41598_2019_55920_MOESM1_ESM.pdf]

Supplementary Figures

for

“Phenotypic alterations in liver cancer cells induced by mechanochemical disruption”

by

Murad HY, Bortz EP, Yu H, Luo D, Halliburton GM, Sholl AB, and Khismatullin DB

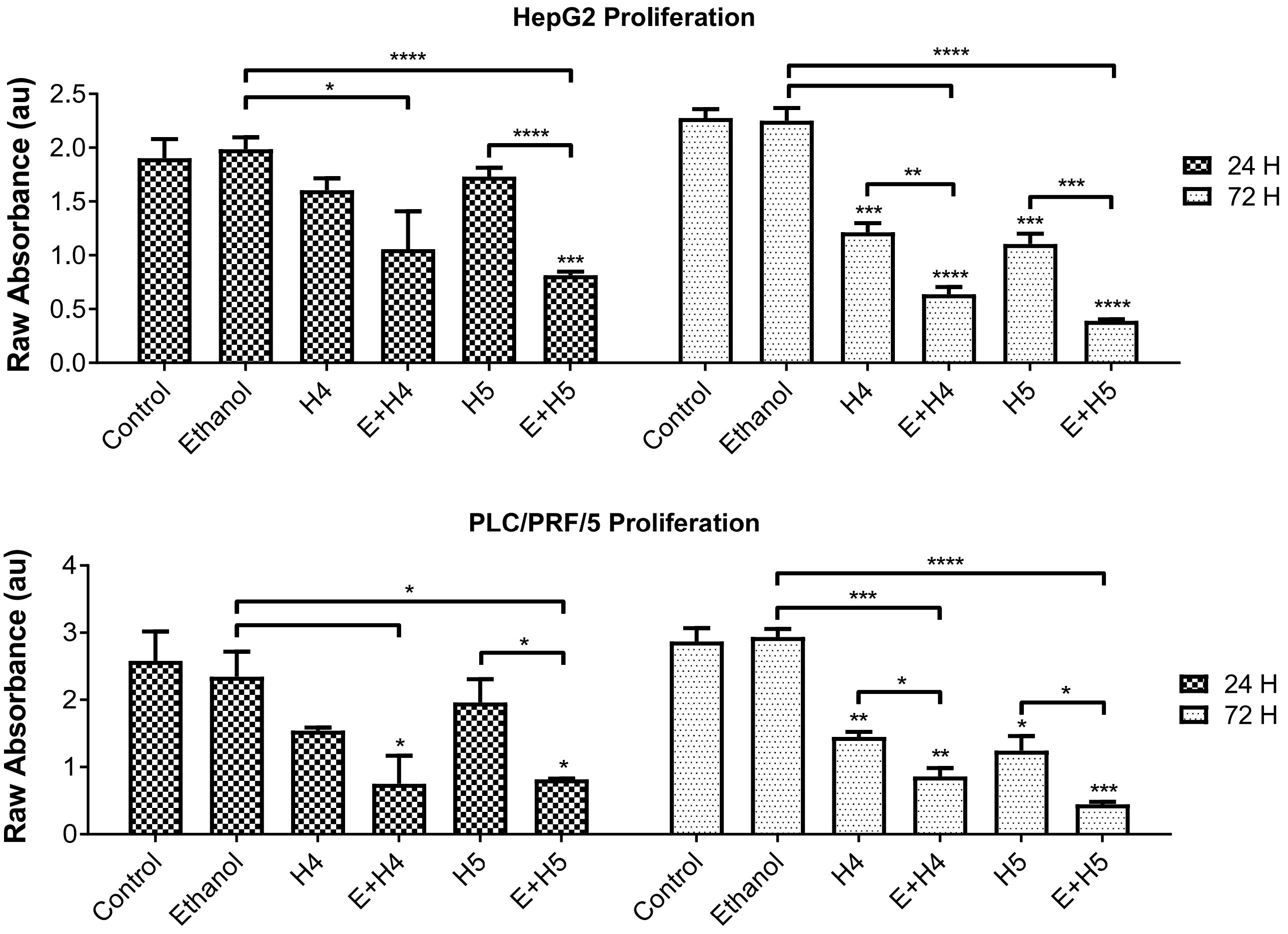

Supplementary Fig. S1. Reduction of proliferation of HCC cells upon exposure to mechanochemical disruption (E+H). HepG2 (top) and PLC/PRF/5 cell proliferation (bottom) was measured by the WST-8 assay at 24 and 72 h post treatment. Values are mean ± SEM of four independent experiments. **p*<0.05, ***p*<0.01, ****p*<0.001, ****p*<0.0001.

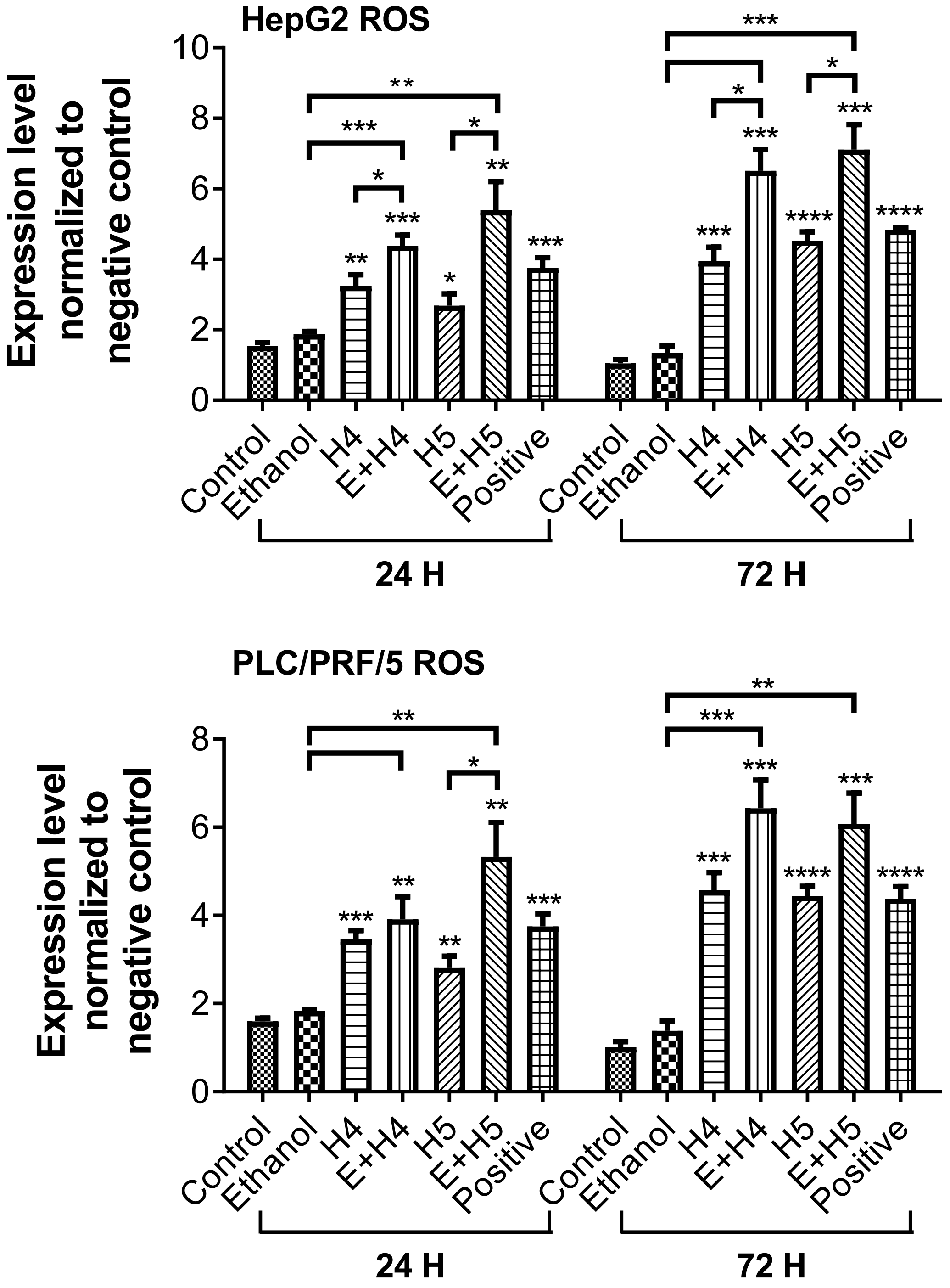

Supplementary Fig. S2. Overproduction of ROS in HCC cells after mechanochemical disruption. ROS production in treated HepG2 (top) and PLC/PRF/5 cells (bottom) was measured by CM-H2DCFDA flow cytometry. Values are mean ± SEM of four independent experiments. **p*<0.05, ***p*<0.01, ****p*<0.001, *****p*<0.0001.

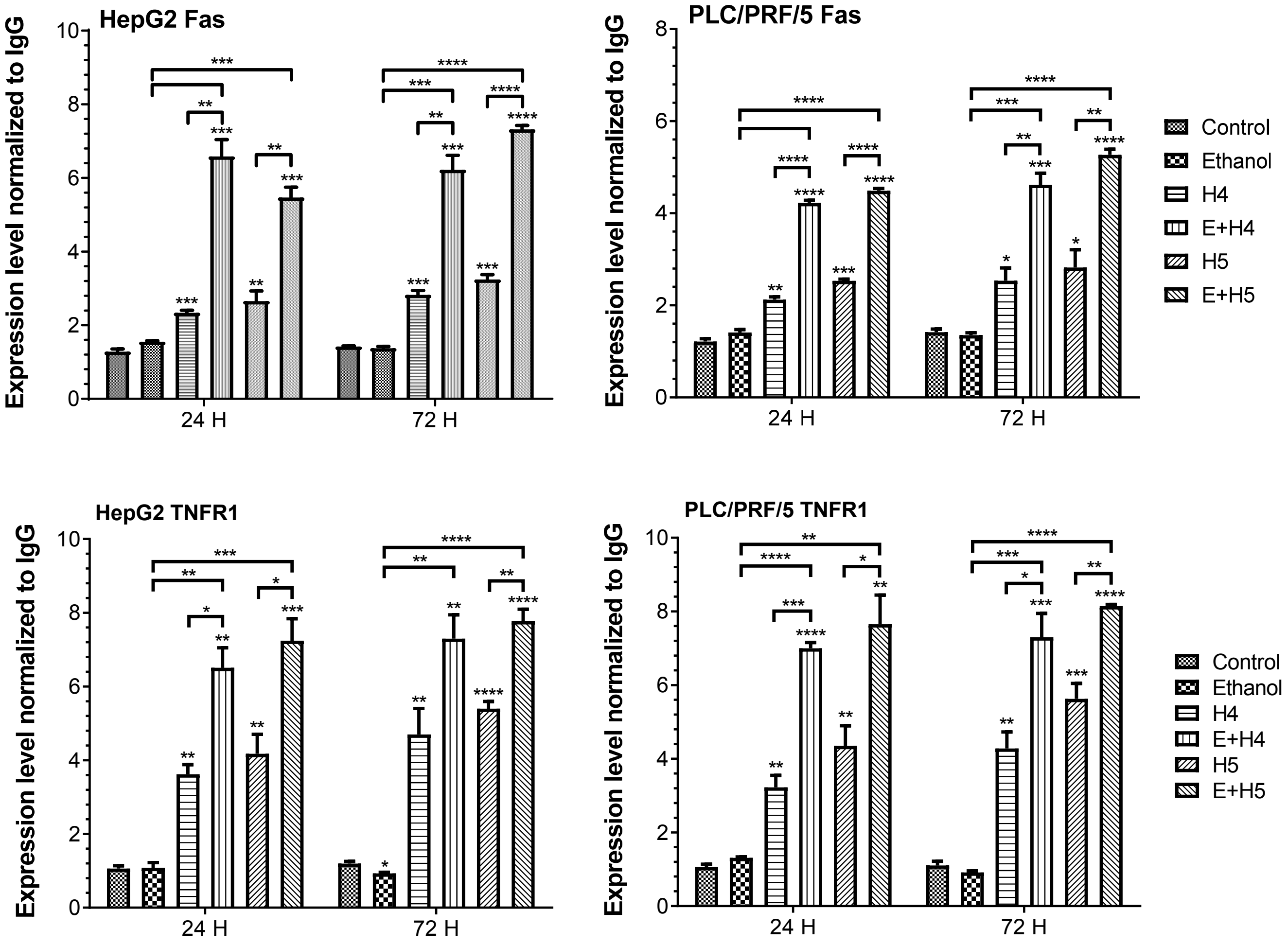

Supplementary Fig. S3. Increased expression and activity of death receptors in HCC cells after mechanochemical disruption. Expression level of Fas (top) and TNFR1 (bottom) in HepG2 (left) and PLC/PRF/5 cells (right), relative to isotype control. Values are mean ± SEM of three independent experiments. *p<0.05, **p<0.01, ***p<0.001, ****p<0.0001.

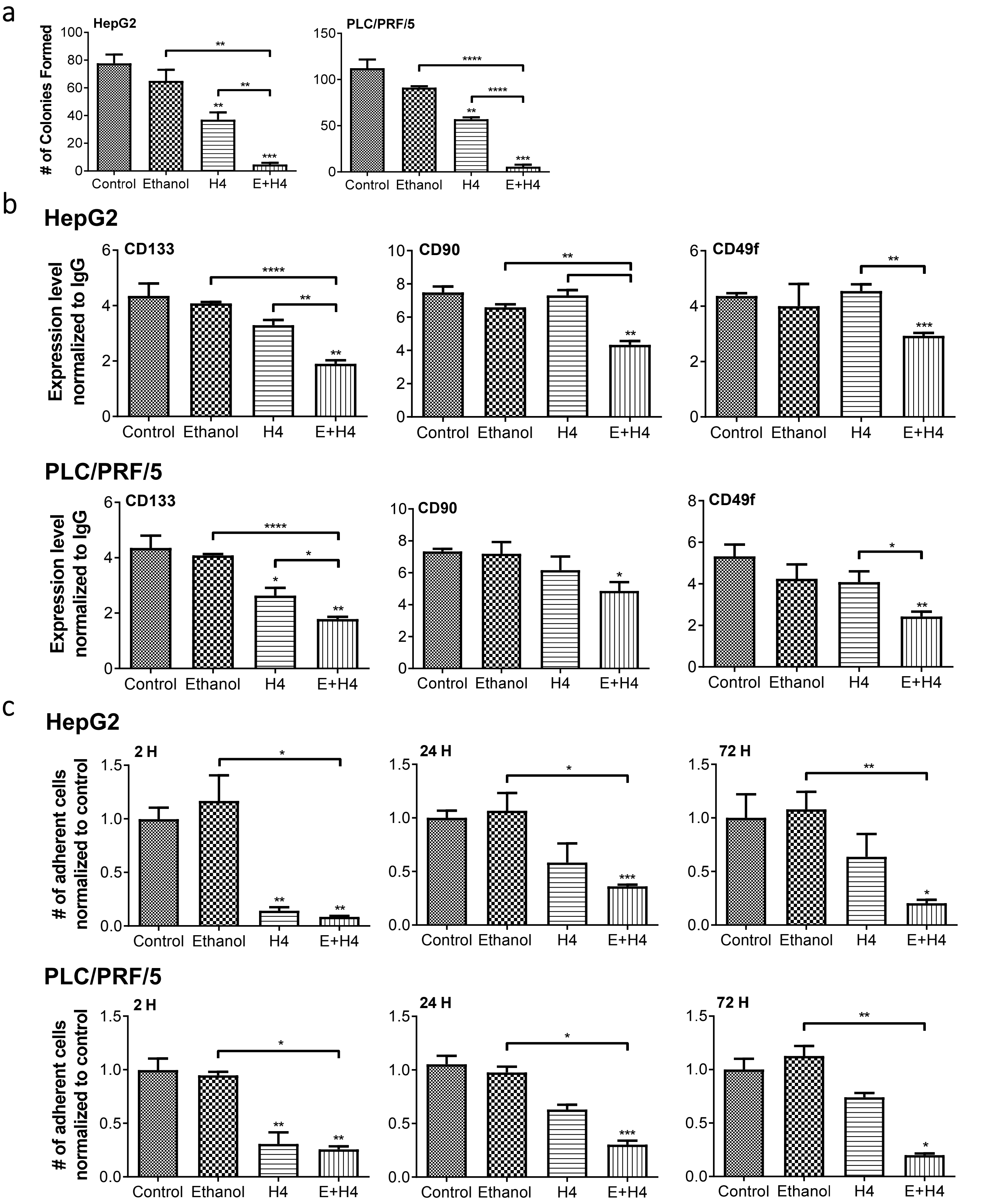

Supplementary Fig. S4. Decreased stemness and adhesion of HCC cells after mechanochemical disruption. (a) Number of HepG2 (left) and PLC/PRF/5 cell colonies (right) formed per treatment group. (b) Normalized-to-IgG expression of CD133 (Prominin 1, left), CD90 (Thy1, middle), and CD49f (α_6 integrin, right) on treated HepG2 (top panel) and PLC/PRF/5 cells (bottom panel). (c) Number of adherent HepG2 (top panel) and PLC/PRF/5 cells (bottom panel) normalized to the control. Values are mean ± SEM of three independent experiments. *p<0.05, **p<0.01, ***p<0.001, ****p<0.0001.

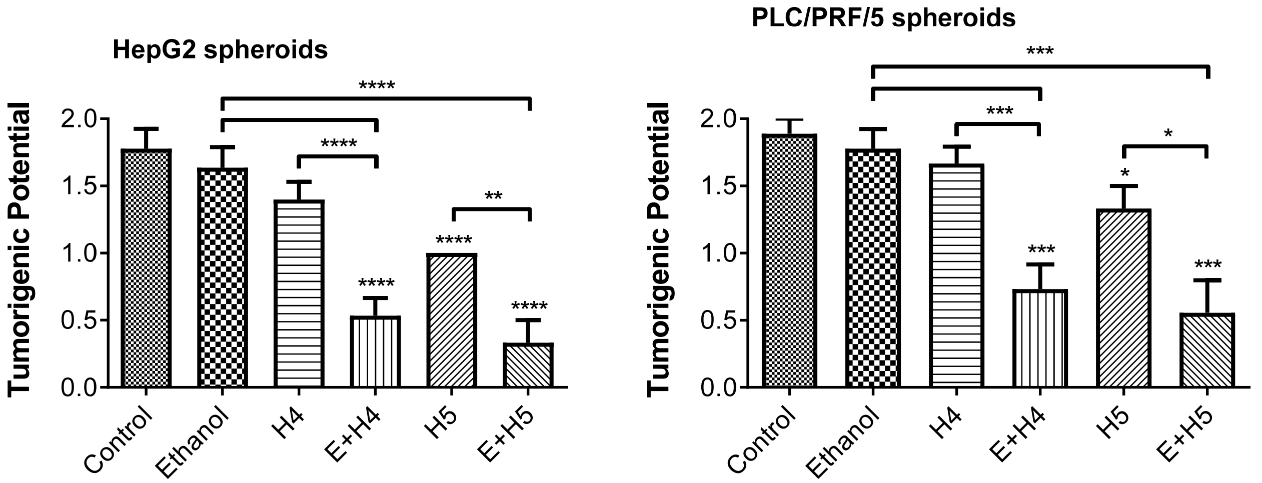

Supplementary Fig. S5. Mechanochemical disruption reduces HCC tumorigenicity *in vitro*. (a) Tumorigenic potential of HepG2 (left) and PLC/PRF/5 cells (right) at day 3 post treatment, assessed based on a 2-point scale, with 0 being no spheroid formation, 1 being loose spheroid formation, and 2 being dense spheroid formation. Values are mean ± SEM of 9-15 independent experiments. **p*<0.05, ***p*<0.01, ****p*<0.001, *****p*<0.0001.
